# Aptamers Selected to Postoperative Lung Adenocarcinoma Detect Circulating Tumor Cells in Human Blood

**DOI:** 10.1038/mt.2015.108

**Published:** 2015-07-07

**Authors:** Galina S Zamay, Olga S Kolovskaya, Tatiana N Zamay, Yury E Glazyrin, Alexey V Krat, Olga Zubkova, Ekaterina Spivak, Mohammed Wehbe, Ana Gargaun, Darija Muharemagic, Mariia Komarova, Valentina Grigorieva, Andrey Savchenko, Andrey A Modestov, Maxim V Berezovski, Anna S Zamay

**Affiliations:** 1 Krasnoyarsk State Medical University named after prof V.F. Voino-Yasenetsky, Krasnoyarsk, Russia; 2 Krasnoyarsk Research Center, Siberian Branch Russian Academy of Sciences, Krasnoyarsk, Russia; 3 Siberian Federal University, Krasnoyarsk, Russia; 4 Krasnoyarsk Regional Clinical Cancer Center named after A.I. Kryzhanovsky, Krasnoyarsk, Russia; 5 Department of Chemistry and Biomolecular Sciences, University of Ottawa, Ottawa, Ontario, Canada

## Abstract

Circulating tumor cells (CTCs) are rare cells and valuable clinical markers of prognosis of metastasis formation and prediction of patient survival. Most CTC analyses are based on the antibody-based detection of a few epithelial markers; therefore miss an important portion of mesenchymal cancer cells circulating in blood. In this work, we selected and identified DNA aptamers as specific affinity probes that bind to lung adenocarcinoma cells derived from postoperative tissues. The unique feature of our selection strategy is that aptamers are produced for lung cancer cell biomarkers in their native state and conformation without previous knowledge of the biomarkers. The aptamers did not bind to normal lung cells and lymphocytes, and had very low affinity to A549 lung adenocarcinoma culture. We applied these aptamers to detect CTCs, apoptotic bodies, and microemboli in clinical samples of peripheral blood of lung cancer and metastatic lung cancer patients. We identified aptamer-associated protein biomarkers for lung cancer such as vimentin, annexin A2, annexin A5, histone 2B, neutrophil defensin, and clusterin. Tumor-specific aptamers can be produced for individual patients and synthesized many times during anticancer therapy, thereby opening up the possibility of personalized diagnostics.

## Introduction

Lung cancer is a leading cause of cancer-related death in the world.^1^ Despite a wide array of investigative methods at the time of diagnosis, the vast majority of patients are in advanced stages (IIIB or IV) of the disease; at which point the cancer is no longer responsive to current therapies.^2^ Early clinical diagnosis of premetastatic malignancy tumors could increase treatment efficiency and survival rate of cancer patients. One perspective tool for better lung cancer diagnosis and monitoring during treatment is evaluation and enumeration of circulating tumor cells (CTCs) and microemboli (CTM) in blood.^3,4^ Besides CTCs and CTM, breakdown products of tumor cells, such as apoptotic bodies (ABs) in blood of cancer patients can be also used for diagnosis. Furthermore, it has been shown that quantifying apoptotic CTCs throughout the course and treatment of the disease has potential for evaluating the response in clinical trials of anticancer therapeutics.^5^

However, the cellular heterogeneity and low abundance of CTCs (<100 cells/ml) in blood represent formidable analytical challenges. Most CTC detection techniques rely on antibody-based capture and staining of cytokeratins and tumor-specific antigens like EpCAM, EphB4, EGFR (Her/ErbB1), HER2, CEA/CEACAM5, and MUC-1.^6^ No single antibody is sufficient to capture and detect all rare CTCs. For instance, EpCAM is not a perfect marker for CTC selection due to the high variation in its gene expression between tumor subtypes and leukocytes.^7^ Many CTCs express epithelial, mesenchymal, and stem-cell markers.

We seek to use DNA aptamers to known and unknown CTC markers. Multiple studies have demonstrated prospective applications of DNA and RNA aptamers for detection of tumor cells..^8,9,10^ Nevertheless, questions are still raised as to whether they can be used for effective CTC detection in clinical blood samples.

In this work, we selected DNA aptamers to lung adenocarcinoma cells derived from postoperative tissues without prior knowledge of protein biomarkers. We specifically chose adenocarcinoma as an example of a highly heterogeneous tumor containing invasive and noninvasive cancer cells. The invasive cells enter blood vessels and become CTCs. Normal lung cells and lymphocytes were used as negative cells for counter-selection of aptamers. We found that the resulting aptamers did not bind to healthy lung cells and A549 lung adenocarcinoma cell line. Additionally, we presented an improved blood preparation technique for the destruction of red blood cells and partial destruction of leukocytes to facilitate the recognition of CTCs, CTM, and ABs.

## Results

### Identification of antilung adenocarcinoma aptamers

**Figure 1:**
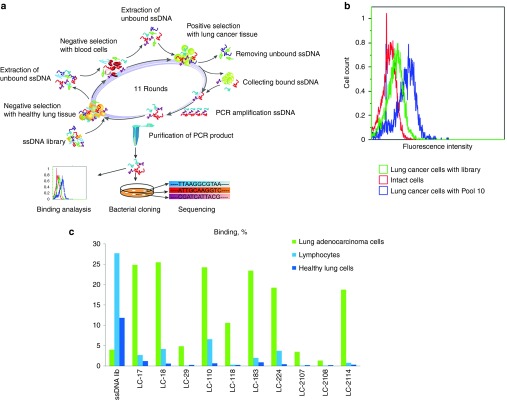
**Aptamer selection to lung adenocarcinoma postoperative tissues**. (**a**) Scheme of cell-SELEX selection of DNA aptamers. (**b**) Flow cytometry binding analysis of 10th pool and the ssDNA library to tumor lung cells. (**c**) The level of aptamer binding to lung-derived tumor cells, healthy cells, and lymphocytes measured by flow cytometry. The ssDNA library was used as a control of nonspecific binding.

Human cancer lung tissues were aseptically taken right after surgery and utilized for an aptamer selection on the basis of cell-SELEX technique.^11,12,13,14,15,16^ The first round included only the positive selection of lung adenocarcinoma cells from postoperative tissues. Healthy lung tissues from distant areas of the same cancer patient and whole blood of a healthy person were used for negative selection steps. A scheme of the aptamer selection procedure is shown in **Figure 1a**. The second and each subsequent round started from negative selection and consisted of the following steps: (i) incubation with healthy lung tissue; (ii) extraction of unbound DNA; (iii) incubation with blood cells from a healthy person; (iv) extraction of unbound DNA; (v) positive selection with lung cancer tissue; (vi) collection of bound DNA; (vii) polymerase chain reaction (PCR) amplification of DNA by symmetric and asymmetric PCR; and (viii) purification of PCR-product from PCR mixture. In total, 11 rounds of selection were performed. The detailed description of the tissue types and blood used for positive and negative selection for each round is provided in **Supplementary Table S1** (**Supplementary Information**). The degree of binding of aptamer pools of different rounds with target cells was assessed by flow cytometry. The 10th aptamer pool with the strongest binding (**Figure 1b**) was cloned in *Escherichia coli* and sequenced.

**Figure 2:**
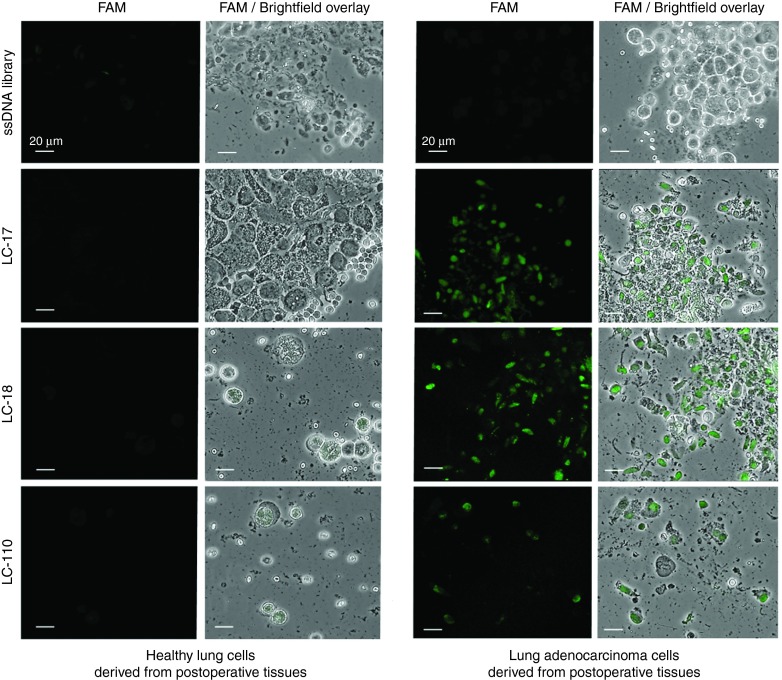
**Staining of healthy lung cells and resected lung adenocarcinoma cells with FAM-labeled aptamer clones (LC-17, LC-18, and LC-110)**. The FAM-labeled ssDNA library was used as a control of non-specific staining. Magnification ×40. FAM, 6-fluorescein amidite.

Thirty-five unique aptamer sequences were synthesized. Ten synthetic aptamers had 5–25-folds higher binding degree to cancer cells isolated from resected tumors than to lymphocytes and lung cells derived from healthy tissues (**Figures 1b** and **2**). DNA sequences of the corresponding aptamers are presented in **Supplementary Table S2**. It is important to note that several aptamer's clones LC-17, 18, 110, 183, and 224 had no or low binding to commercially available adenocarcinoma cell line A549 (**Supplementary Figure S1**).

**Figure 3:**
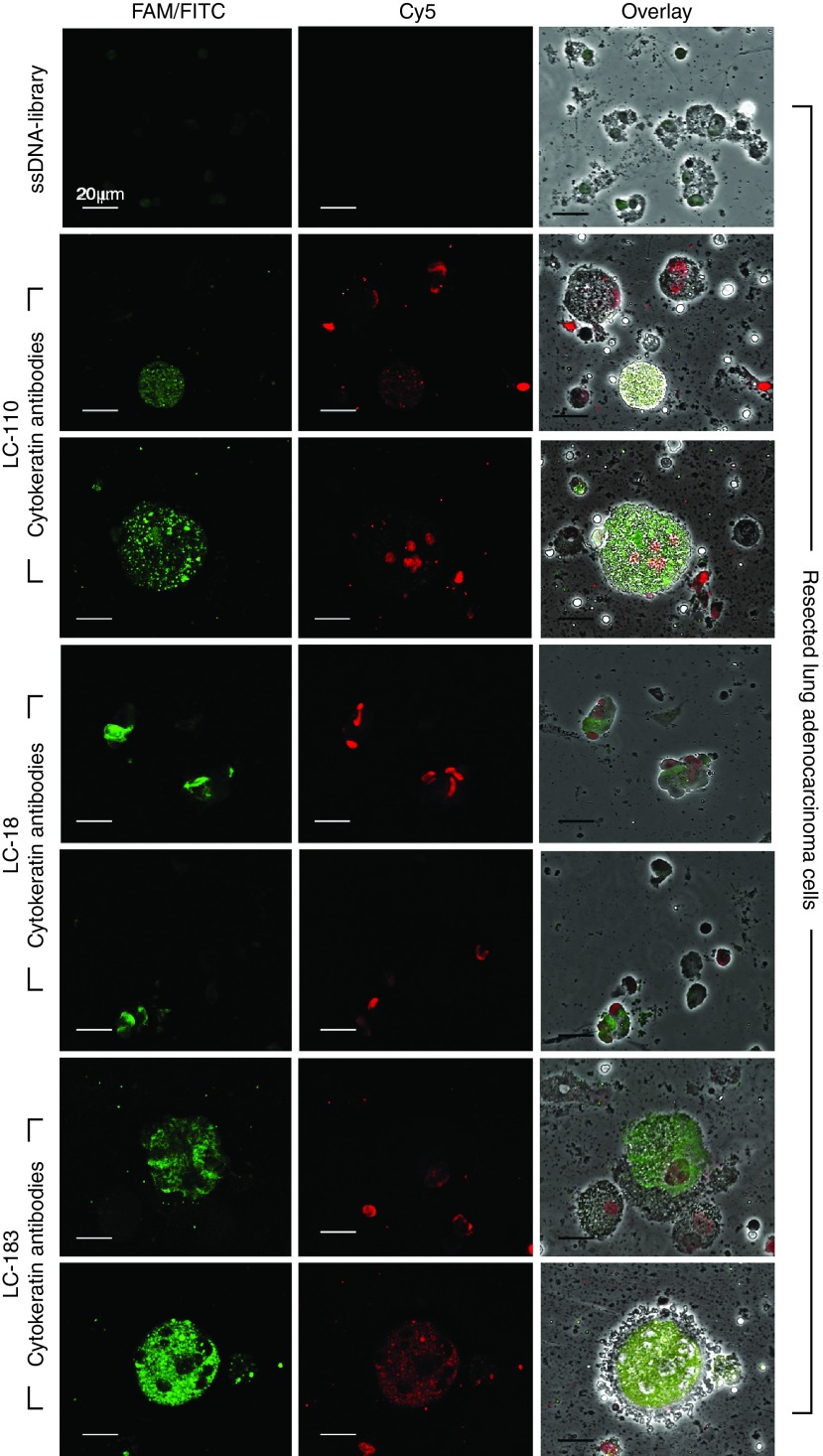
**Costaining of lung adenocarcinoma cells with Cy-5-labeled aptamers (LC-18, LC-110, LC-183) and FITC-labeled anti-pan cytokeratin antibodies**. Magnification ×60.

To validate aptamer binding to cancer cells, we observed lung adenocarcinoma cells costained with Cy5-labeled LC-18, LC-110, LC-183 aptamers and fluorescein isothiocyanate (FITC)-labeled anti-pan cytokeratin antibodies in confocal microscopy experiments (**Figure 3**). We found that these aptamers recognized different epitopes than anti-pan cytokeratin antibodies.

### Identification of aptamer-associated protein biomarkers of adenocarcinoma

We identified aptamers' protein targets using an Aptamer-facilitated Biomarker Discovery technique (AptaBiD).^17,18^ In these experiments, we used tumor tissues from three lung cancer patients. The target proteins were captured and purified from minced lung tumor tissues using aptamer-coated magnetic particles and identified using high-performance liquid chromatography and high-resolution tandem mass spectrometry. Potential protein targets were ranked according to the correlation coefficient calculated based on the relative abundance of a protein in an aptamer-isolated sample versus the abundance of the same protein in a control sample isolated using a DNA library (**Supplementary Tables S3**–**S5**). The most probable protein targets for the aptamers from all three patients are presented in **Table 1**. For LC-183 aptamer, a potential target was cathepsin D. This protein is involved in the degradation of intracellular proteins, hormones, growth factors, and receptors. This protein is known to be overexpressed in lung, breast, and colorectal tumors^19,20,21^ and can be one of the prognostic factors for the cancers.

In addition, we identified potential protein hits such as: vimentin, annexin A2, and annexin A5 for LC-17 aptamer, histone H2B and neutrophil defensin for LC-18 aptamer, and clusterin and histone H2B for aptamer LC-110.

### Detection of CTCs in blood

**Figure 4:**
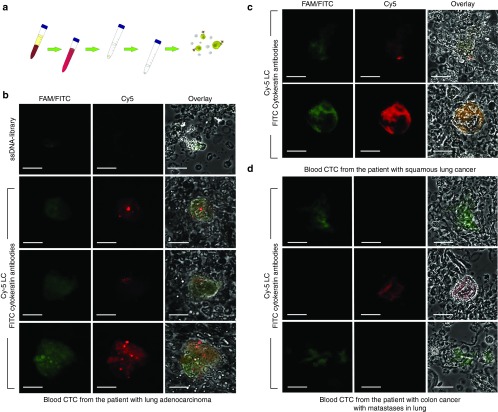
**Circulating tumor cell (CTC) identification in clinical blood samples**. (**a**) Blood sample preparation. Red blood cells were lysed with hypotonic NH_4_Cl solution followed by incubation with hypotonic NaCl. (**b**) Costaining of CTCs from blood of a patient with lung adenocarcinoma by a Cy-5-labeled LC-18 aptamer and FITC-labeled anti-pan cytokeratin antibodies. (**c**) Costaining of CTCs from blood of a patient with squamous lung cancer by Cy-5-labeled LC-18 aptamer and FITC-labeled anti-pan cytokeratin antibodies. (**d**) Costaining of CTCs from blood of a colon cancer patient with lung metastases by Cy-5-labeled LC-18 aptamer and FITC-labeled anti-pan cytokeratin antibodies. Magnification ×60.

The blood preparation procedure was improved in order to reduce time and labor required for the search of rare CTCs (**Figure 4a**). All red blood cells were lysed with NH_4_Cl solution and the majority of white blood cells were lysed with hypotonic NaCl solution. The pellet was stained with fluorescently labeled LC-18 aptamer and anti-pan cytokeratin antibodies. The treated blood samples were analyzed by confocal fluorescent microscopy, and tumor elements, presumably CTCs, were found in patients with lung adenocarcinoma (**Figure 4b**), squamous lung cancer (**Figure 4c**) and primary colon cancer with metastases in lung (**Figure 4d**). Interestingly, LC-18 aptamer did not recognize cytokeratin-positive CTCs in blood of a patient with colon cancer with metastases in lung.

**Figure 5:**
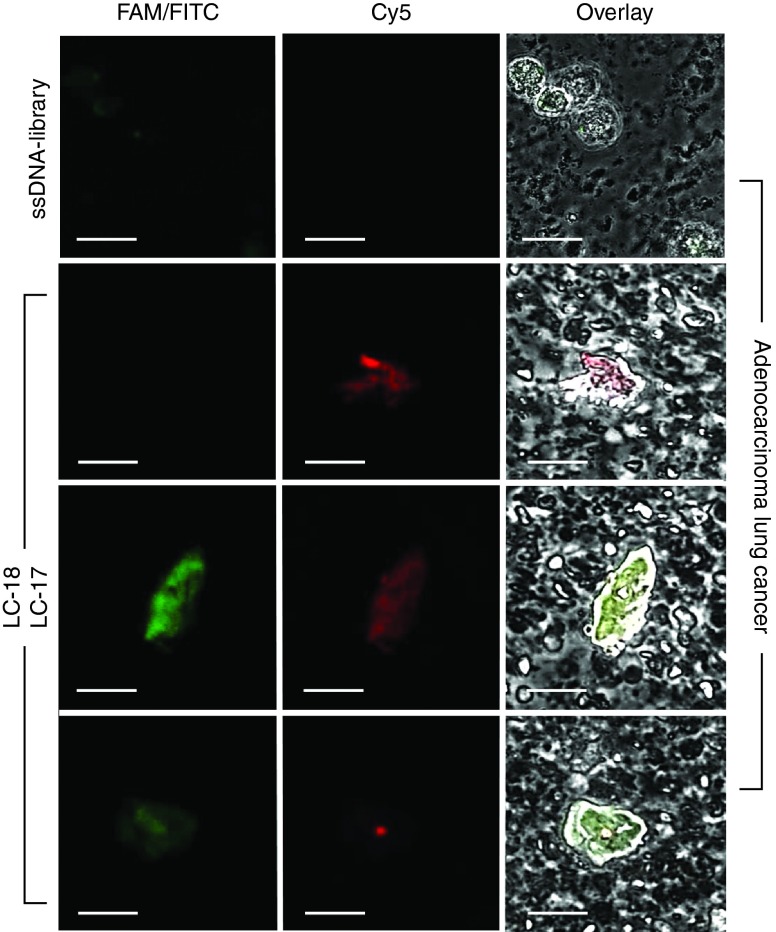
**Costaining of lung adenocarcinoma cells with two different aptamer clones, Cy5-labeled LC-18 and FAM-labeled LC-17**. Magnification ×60.

We also tested another aptamer clone, LC-17. It did bind to two of the three CTCs whereas LC-18 bound to all tumor cells as shown in **Figure 5**. Nuclei staining with DNA intercalating dyes could be helpful for the better identification of individual cells and interpretation of results. The problem is that aptamers will be also stained with the dyes and give high background fluorescence. Therefore, we applied Romanowsky-Giemsa staining on fixed smears that allows seeing the nuclei and aptamers. For these analyses, 3 ml of vein blood were taken and CTCs were concentrated the same way as for the fresh blood staining. Blood smears stained with LC-18 aptamer and Romanowsky-Giemsa dye provided further information about different tumor elements, because the combined staining could distinguish CTCs, apoptotic bodies, and microemboli. After treating with masking RNA and staining with the aptamers as described in Materials and Methods, the smears of blood were uniformly deposited on glass slides fixed with methanol and stained with Romanowsky-Giemsa dye.

**Figure 6:**
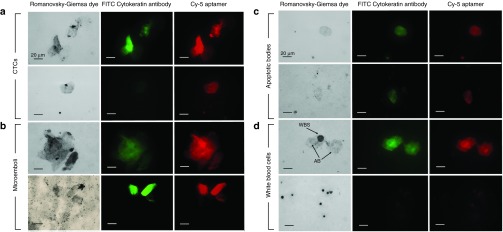
**Fluorescent microscopy of blood smears of lung adenocarcinoma**. Samples were preincubated with masking RNA, Cy-5-labeled LC-18 and FITC-labeled anti-pan cytokeratin antibodies. The samples were spread evenly on a glass slide. The smears were fixed in methanol for 5 minutes and then stained with Romanowsky-Giemsa dye. Magnification ×60.

**Figure 6** represents tumor elements from the blood of lung cancer patients costained with the aptamers, cytokeratin antibody, and Romanowsky-Giemsa. Tumor elements morphology could be evaluated in the brightfield (left panel) and fluorescent view (middle and right panels are for cytokeratin and aptamer staining). In **Figure 6** **(panels a and b)**, CTCs and microemboli can be seen; in panel **c** of **Figure 6**, the tumor elements have no nucleus and were less stained with Romanowsky-Giemsa dye, which indicated that it was likely an apoptotic body. Panel **d** of **Figure 6** demonstrates how white blood cells look comparing with apoptotic bodies.

We have analyzed blood smears from 105 individuals: 18 healthy people and 87 patients with various diagnosis including different types of primary lung cancer (52), secondary lung cancer (1), other lung diseases (9), breast diseases (9), and glioblastoma (16) (**Table 2** and **Supplementary Figures S2 and S3**). Identification by aptamers as well as cytokeratin antibodies reviled 15 patients out 52 with proved primary lung cancer had less then 2 cells per 3 ml of blood, and 8 of them did not have CTCs and microemboli at all. Aptamers appeared to be more sensitive to CTCs than cytokeratin antibodies; in some cases, more aptamer-positive cells were found in the sample comparing with cytokeratin antibodies. Two patients with lung adenocarcinoma did not have cytokeratin-positive CTCs at all, but these cells were stained with aptamers. False negative results were registered in six cases. Two of them had lung diseases: hondrogamartoma (nine CTCs were detected) and tuberculosis (four cells); another two had breast diseases: cystitis adenopapilloma (two cells), adenocarcinoma (four cells and two microemboli were aptamer-positive and seven cells and 0 microemboli were cytokeratin-positive); and two patients with glioblastoma had five and two cells in 3 ml of blood.

Based on these results, we have calculated the sensitivity and specificity of the method of CTC detection using blood smears stained with the aptamers to lung cancer. If two CTCs/microemboli or more were found, we considered it as a positive result.

Sensitivity: Se = (TP/(TP+FN)) × 100% = (37/(37+6)) × 100% = 86%

Specificity: Sp = (TN/(TN+FP)) × 100% = (48/(48+15)) × 100% = 76%

Where TP is true positive (lung cancer is correctly diagnosed), FN is false negative (lung cancer is incorrectly identified in blood of healthy people or people with other diseases), TN is true negative (healthy people and people with other diseases correctly identified as they do not have lung cancer), and FP is false positive (people with lung cancer incorrectly identified as healthy). Thus, the aptamer method could be used as an additional diagnostic tool for estimating invasiveness and monitoring the course of the tumor treatment and development.

## Discussion

Dissemination of tumor cells through the bloodstream is a key step in understanding the progression of solid tumors such as lung cancer.^22^ Therefore, measuring a tumor cell level in blood can be used for noninvasive diagnosis of lung cancer as well as for the estimation of treatment efficacy.^3^

The most common CTC markers are cytokeratins and epithelial cell adhesion molecules (EpCAM).^4^ However, finding additional CTC markers and corresponding probes are in great demand due to high tumor diversity. The unique feature of our approach is that we select synthetic affinity probes (aptamers) to cancer cells biomarkers in their native state and conformation without previous knowledge of biomarkers. The biomarkers are identified after the aptamer selection by aptamer-mediated isolation with magnetic beads and following mass spectrometry-based analysis.

To date, most of the aptamers have been selected to cell cultures of several histological types of lung cancer.^11,13,23^ However, such aptamers are often specific only to surface proteins of cultured cancer cells and may not bind to tumor cells in clinical samples because of the difference in protein expression between cultured tumor cells and primary tumor cells. In our study, aptamers were selected to clinical tissue samples of lung adenocarcinoma. In order to obtain aptamers with greater potential for detection of tumor-specific elements, we used the postoperative human tissue samples for each round of aptamer selection.

Here, we showed that aptamers could detect CTCs in clinical blood samples in which the majority of the erythrocytes and leukocytes were lysed with hypotonic solutions. The aptamers were found to bind to cytokeratin-positive and negative tumor elements in the blood of patients with lung adenocarcinoma and squamous cell carcinoma (**Figure 4**). It has been shown that metastases that occur in other organs represent a clonal population of the primary tumor, but genetically evolved from the original parental clone.^24^ This means that aptamers selected to postoperative lung cancer tissues may have the ability to bind to metastatic lung tumor cells circulating in blood.

Our confocal microscopy analysis confirmed that aptamers specifically bind to different biomarkers than anti-pan cytokeratin antibodies (**Figures 3** and **4**) and can be used together with antibodies. Moreover, cancer cells circulating in blood can lose cytokeratin^25^ and indeed in some cases we saw cells in circulation that were not recognized by anti-pan cytokeratin antibodies but stained with a Cy-5-labeled aptamer (**Figure 3**).

Due to the binding of multiple aptamers to different cell biomarkers, a pool of tens and hundreds of aptamer clones will be more selective and efficient in CTC detection, than a single aptamer or a monoclonal antibody. Application of aptamers in combination with antibodies to tumor-specific antigens will provide more reliable detection of rare CTCs. Such tumor-specific aptamers can be produced for individual patients and synthesized many times during anticancer therapy, thereby opening up the possibility of personalized diagnostics.

## Materials and Methods

***Materials.*** All deoxyoligonucleotides were purchased from Integrated DNA Technologies (IDT, Coralville, Iowa). Streptavidin MagneSphere Paramagnetic Particles were purchased from Promega Corporation (Madison, WI), iodoacetamide from Pierce Biotechnology (Rockford, IL), and ready-to-go pipette tips ﬁlled with C18 spherical silica reversed-phase material ZipTipC18 from Millipore (Etobicoke, Canada). All other reagents, media and buffers and antibodies were purchased from Sigma-Aldrich (Oakville, Canada), unless otherwise stated.

This study was approved by the Local Committee on Ethics of the Krasnoyarsk Regional Clinical Cancer Center named after A.I. Kryzhanovsky and Krasnoyarsk State Medical University, Krasnoyarsk, Russia. Tumors for this study were taken from patients who had undergone complete, curative resection of their tumors. The lung cancer specimens were collected with the written informed consent of patients. Solid tumors were removed aseptically and immediately immersed in ice-cold colorless Roswell Park Memorial Institute (RPMI)-1640 supplemented with 1,000 U/ml penicillin G and 1,000 mg/l streptomycin. Samples were transported on ice to the laboratory for aptamer selection and primary cell culturing within 1 hour of collection.

***Preparation of lung cancer cells from resected samples.*** Solid tumor specimens were rinsed twice with Dulbecco's Modified Eagle's medium (DMEM) supplemented with 100 U/ml penicillin G and 100 mg/l streptomycin, necrotic tissues, fatty tissues, blood clots and apparently normal tissues were dissected. Tissues were minced with scissors and dissociated into small aggregates by pipetting. Heavy pieces sedimented by gravity were removed. Tumor cell aggregates were collected and washed with Dulbecco's phosphate-buffered saline (DPBS) several times after the last centrifugation; the pellet was resuspended in incomplete AR-5 initiation medium (ACL-4 with 5% fetal bovine serum), transferred to 25-cm^2^ flasks, and maintained in humidified incubator at 37 °C in an atmosphere of 5% CO2 and 95% of air. ACL-4 medium is formulated for selective growth of human lung adenocarcinoma and contains 20 µg/ml insulin, 10 µg/ml transferring, 50 nmol/l hydrocortisone, 1 ng/ml epidermal growth factor, 100 pmol/l triiodothyronine, 10 µmol/l ethanolamine, 2 mmol/l glutamine, 0.5 mmol/l sodium pyruvate, 2 mg/ml bovine serum albumin, 25 nmol/l sodium selenite, 100 U/ml antibiotics, and 25 mmol/l HEPES buffer (to compensate the loss of buffering properties) added to a basal RPMI-1640 medium. The next day, all floating cells were removed, and the fresh medium added was changed once or twice a week. A cell scraper was used to remove visible fibroblast growth whenever it occurred. Once the cells were confluent, they were digested with 0.05% Ethylenediaminetetraacetic acid (EDTA)-trypsin for passage. After two passages, the cells were taken for the experiments.

***Aptamer selection.*** Aptamers were selected on the basis of the cell-SELEX procedure. The selection started with a single-stranded DNA library (Integrated DNA Technologies) which consisted of a randomized region of 40 nucleotides (N40) flanked by two constant primer-hybridization sites (5′-CTC CTC TGA CTG TAA CCA CG N40 GC ATA GGT AGT CCA GAA GCC-3′). Before each round of selection and binding experiment, the DNA library and aptamer pools were denatured by heating for 5 minutes at 95 °C in Dulbecco's phosphate-buffered saline (Sigma-Aldrich) and then renatured on ice for 10 minutes. Healthy lung tissue of a patient and whole blood of a healthy person were used for negative selection steps.

The first round of selection omitted the negative selection step. Thus, lung adenocarcinoma cells derived from fresh tumor tissue samples were incubated in 500 µl DPBS containing 1 µmol/l (0.1 nmol or 6 × 10^13^ sequences) DNA library for 30 minutes at 25 °C and then centrifuged at 3,500 g for 10 minutes at 25 °C to remove unbound aptamers, followed by washing twice with DPBS. The pellet was then resuspended in 95 µl of 10 mmol/l Tris-HCl buffer containing 10 mmol/l EDTA, pH 7.4 (TE) (Sigma-Aldrich) and heated for 10 minutes at 95 °C to release aptamers bound to the lung adenocarcinoma cells. After the denaturing step, debris was removed by centrifugation at 14,000 g for 15 minutes at 4 °C and the supernatant (containing the aptamers) was collected and stored at −20 °C. Subsequently, adenocarcinoma-binding aptamers were amplified using symmetric and asymmetric PCR. For symmetric PCR, 5 µl of the aptamer pool in TE was mixed with 45 µl of symmetric PCR Master Mix containing: 1× PCR buffer, 2.5 mmol/l MgCl_2_, 0.025 U/μl KAPA2G HotStart Robust polymerase (KAPABiosystems), 220 µmol/l an equimolar solution of dATP, dCTP, dGTP and dTTP (dNTPs), 300 nmol/l forward primer (5′-CTC CTC TGA CTG TAA CCA CG-3′), and 300 nmol/l reverse primer (5′-GGC TTC TGG ACT ACC TAT GC-3′). Afterward, asymmetric PCR was performed, where 5 µl of the symmetric PCR product in TE buffer was mixed with 45 µl of the asymmetric PCR Master Mix containing: 1× PCR buffer, 2.5 mmol/l MgCl_2_, 0.025 U/μl KAPA2G HotStart Robust polymerase, 220 µmol/l dNTPs, 1 µmol/l forward Cy5 primer (5′-Cy5- CTC CTC TGA CTG TAA CCA CG-3′), and 50 nmol/l reverse primer (5′- GGC TTC TGG ACT ACC TAT GC-3′). Amplification was performed using the following PCR program: preheat for 2 minutes at 95 °C, 15 cycles of 30 seconds at 95 °C, 15 seconds at 56 °C, 15 seconds at 72 °C, and hold at 4 °C.

The concentration of PCR products was estimated by separation of Cy5-labeled DNA on 3% agar electrophoresis. Agar gels were analyzed in the gel-documenting system GBOX/EF2-E, which allowed examining aptamer purity and concentration. An aptamer pool was stored at −20 °C for subsequent rounds of selection. In negative selection, heparinized whole blood from a healthy person and cells derived from distant healthy tissues of the same patient in DPBS were used. The cell mixture was incubated for 30 minutes at 25 °C in 500 µl DPBS with 100 nmol/l aptamer pool from a previous round and then centrifuged at 3,500 g for 10 minutes at 25 °C to collect unbound sequences. The unbound aptamers were used for following positive selection as described above. An aptamer pool was stored at −20 °C and used for the next round of selection following the aforementioned procedure, starting from negative selection. The concentration of aptamers was determined with a NanoVue plus spectrophotometer (GE Healthcare, Life Sciences, Moscow, Russian Federation).

In total, 11 rounds of selection were performed. Affinity of aptamers to adenocarcinoma cells was evaluated by flow cytometry using a FC-500 Flow Cytometer (Beckman Coulter, Moscow, Russian Federation). The best pool was cloned using a M13mp18 Perfectly Blunt Cloning Kit (Novagen, Germany) according to the manufacturer's protocol. Aptamer clones were sequenced at the Génome Québec Innovation Centre (Canada). Aptamer clones were synthesized at Integrated DNA Technologies. All further studies were performed with the synthetic aptamer clones.

***Flow cytometric binding analysis of aptamers.*** The binding and specificity of the evolved aptamers was evaluated by flow cytometry using FC-500 Flow Cytometer (Beckman Coulter). Lung adenocarcinoma material was minced into small pieces and then pipetted gently with DPBS to remove cell clusters and obtain a homogeneous solution. Thereafter, obtained cells were centrifuged at 3,500 g for 5 minutes and washed with DPBS. Cells were preincubated with masking DNA (0.1 mg/ml of salmon sperm DNA) for 30 minutes and then with 50 nmol/l of Cy5-labeled aptamers from pools 7 to 11 of selection rounds. Each sample contained 1 × 10^5^ cells. The DNA library was used as a control. The measurements were carried out using flow cytometry for 30,000 events.

***Confocal microscopy binding analysis of aptamers.*** Confocal microscope Olympus Fluoview 10vi (Olympus Optical, Japan) was used for all confocal microscopy binding analysis studies and all photos were processed with CELL^F^ Imaging Software for Life Science Microscopy. Lung adenocarcinoma cells, healthy lung cells isolated from the postoperative tissues according to the procedure described above, A549 cultured cells, were preincubated with 0.1 mg/ml yeast RNA as a masking single-stranded nucleic acid for 20 minutes at 25 °C and than with 50 nmol/l LC-18, LC-17, LC-110 Cy5-labeled aptamers or FAM-labeled library for 30 minutes at 25 °C, washed with DPBS once, centrifuged at 3,500 g for 5 minutes and analyzed the pellet under the confocal microscope.

***Identification of tumor components in clinical blood samples.*** Blood samples (total volume 3 ml) for the study were drawn from the vein of patients with the addition of an anticoagulant. Red blood cells were lysed with hypotonic NH_4_Cl solution followed by centrifugation at 3,500 g for 5 minutes. Subsequently, the majority of the white blood cells were incubated with hypotonic NaCl solution for one hour followed by centrifugation at 3,500 g for 5 minutes. The pellet was incubated with 0.9% NaCl for 10 minutes, centrifuged and washed twice as described in details in **Supplementary Information**. Next, the pellet was stained with Cy5- or FAM-labeled aptamers LC-18, 17, 29, 224, 2107, 2108, or 2114 and/or FITC-labeled cytokeratin antibodies and analyzed under the confocal microscope as described above. To prepare the smears, the cell pellet after staining was spread evenly on a glass slide and then fixed in methanol for 5 minutes, followed by staining with Romanowsky-Giemsa dye.

***Identification of protein biomarkers.*** One million cells were preincubated for 30 minutes with 1 µmol/l of masking DNA and 100 nmol/l of unlabeled ssDNA library in DPBS. Afterward, incubation continued for another 30 minutes with 50 nmol/l biotinylated aptamer or 50 nmol/l biotinylated ssDNA library as a control. Subsequently, 1 mg of Streptavidin MagneSphere Paramagnetic Particles (Promega Corporation) preincubated at 25 °C with masking DNA was added, and incubated for another 30 minutes with gentle shaking. Cells were lysed with 0.1% (v/v) sodium deoxycholate in DPBS at 25 °C for 30 minutes prior to washing. Protein targets were dissociated from aptamer-coated beads by adding 30 µl of 8 M urea solution and incubated for 1 hour at 25 °C. Magnetic particles were retained using a magnetic stand and the supernatant was removed and stored at −20 °C. Protein concentration in samples was measured using NanoVue plus spectrophotometer (Life Sciences).

A fraction of 10 µl of denatured proteins was diluted with 15 µl of 50 mmol/l ammonium bicarbonate (Sigma-Aldrich) and 1.5 µl of 100 mmol/l dithiothreitol (Sigma-Aldrich), incubated for 1 hour at 37 °C, mixed with 3 µl of 100 mmol/l iodoacetamide (Pierce Biotechnology), and incubated for 30 minutes at 25 °C in the dark. The samples were then diluted with 15 µl of 50 mmol/l ammonium bicarbonate to lower the concentration of urea. Proteins in each sample were digested with porcine trypsin (Sigma-Aldrich) for 16 hours at 37 °C by adding 2 µl of 100 ng/μl trypsin. The peptide mixture was desalted using ready-to-use pipette tips filled with C18 spherical silica reversed phase material (ZipTipC18, Millipore), according to the manufacturer's protocol. Peptides were eluted with 10 µl of 80% acetonitrile and 0.1% trifluoroacetic acid, evaporated with ScanVac (LaboGene ApS, Lynge, Denmark), and resuspended with 10 µl of 0.1% formic acid.

Shotgun mass spectrometric analysis of 5 µl of protein-digest sample was performed by nanoflow ultra high-pressure liquid chromatography (Easy-nLC 1000, Thermo Scientific, Odense, Denmark) and tandem mass spectrometry with an Orbitrap Velos Pro mass spectrometer (Thermo Scientific, Bremen, Germany). Full scan spectra were obtained using a Fourier-transform mass analyzer with a resolution of 60,000. MS spectra of top 15 precursors fragmented by collision-induced dissociation were obtained by an ion trap as a mass analyzer. At the beginning and at the end of experimental sequences, BSA standard digest analyses were performed to estimate the quality of evolved results. Accuracy deviations for precursors were less than 5 ppm, which is appropriate for the external calibration standards. Database searches were done with Proteome Discoverer 1.3 software, Sequest search engine and SwissProt database, and the label-free quantitative analyses were performed using MaxQuant 1.3.0.5 proteomic software. Triplicates of each experiment were performed. Results from independent experiments were analyzed separately. We analyzed quantitatively the protein distributions for each sample. Proteins that were present in the negative control groups (the biotin-labeled DNA library instead of the aptamer and DNA free group), in equal or larger amount than in aptamer samples, were excluded from the list. The proteins were considered reliable when determined by 3 or more peptides. We consider a protein as a potential target for the aptamers when it quantitatively dominated over the samples with the control library in three independent experiments.

**SUPPLEMENTARY MATERIAL** **Figure S1.** Analyses of aptamer specificity to A549 cancer cell line. **Figure S2.** Fluorescent microscopy of blood smears of different lung cancer types. **Figure S3.** Fluorescent microscopy of blood smears of different not lung cancer other diseases and healthy people. **Table S1.** Characteristics of the tissues/blood/patients utilized at each round of aptamer selection to lung adenocarcinoma. **Table S2.** Sequences of individual aptamers selected to postoperative lung adenocarcinoma tissues. **Table S3.** Potential protein targets for aptamer LC-17. **Table S4.** Potential protein targets for the aptamer LC-18. **Table S5.** Potential targets for aptamer LC-110. **Supplementary Information**

## Figures and Tables

**Table 1:**
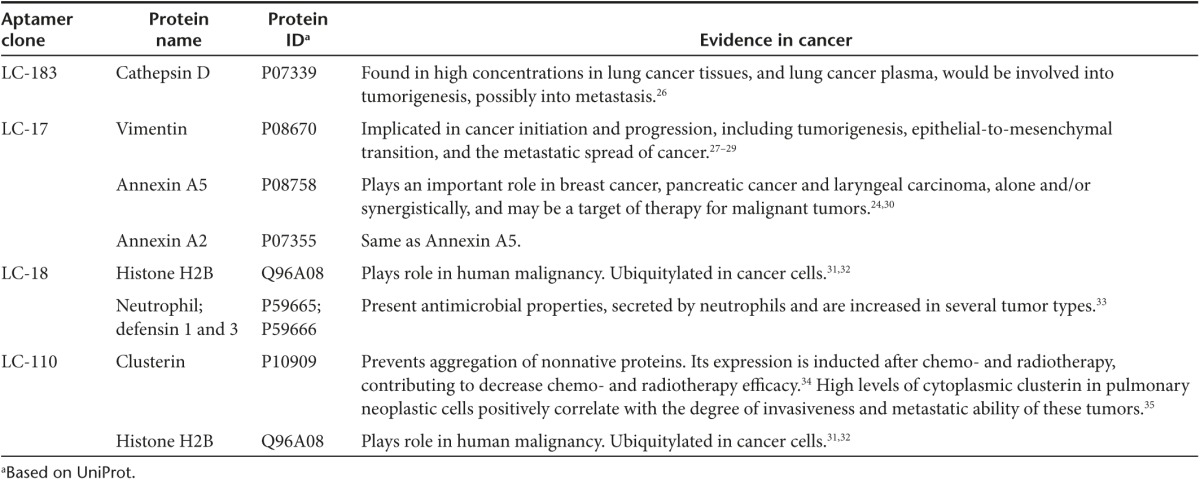
Candidate biomarkers associated with antilung cancer aptamers

**Table 2:**
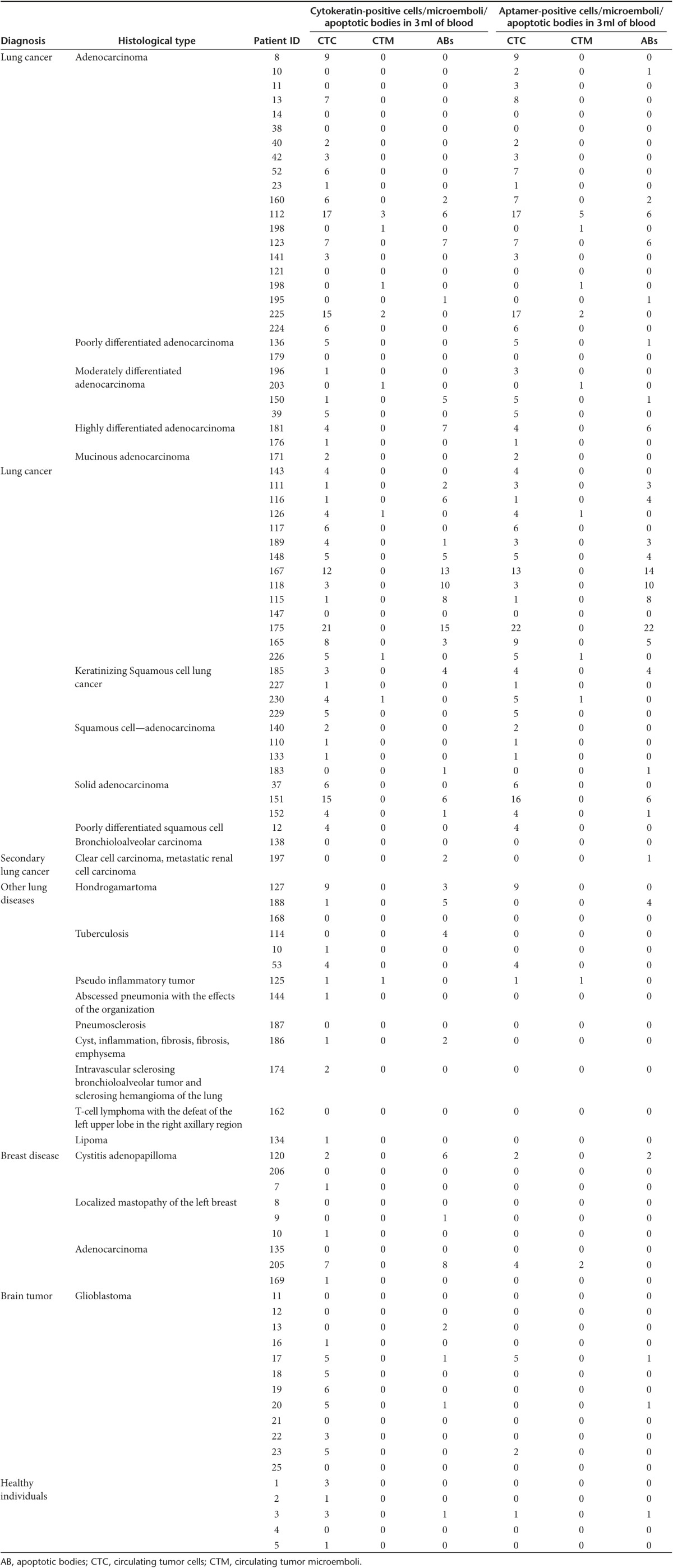
The initial set of statistics for the detection of CTC, CTM, and ABs in 3 ml blood samples of patients with lung cancer, nonmalignant lung diseases, diseases of the breast, lung, brain tumors, dementia, and apparently healthy people using Cy-5 labeled aptamer pool combined from clones LC-17, 29, 224, 2107, 2108, 2114 and FITC-labeled anti-pan cytokeratin antibodies
