# Supplementary material for: Aptamers Selected to Postoperative Lung Adenocarcinoma Detect Circulating Tumor Cells in Human Blood
Source: Mol Ther. 2015 Jul 7;23(9):1486–96. doi: 10.1038/mt.2015.108 (PMC4817883; doi:10.1038/mt.2015.108)
Supplement: Supplementary Information [file mt2015108x1.doc]

**Supplementary Information**

**Detailed protocol for isolation of circulating tumor cells (CTC) from blood and staining with aptamers and Romanowsky-Giemsa dye:**

**List of reagents and materials:**

1. 0.42% w/w NH_4_Cl with heparin (50 units per 100 ml)

2. 0.2% NaCl with heparin (50 units per 100 ml)

3. 0.9% NaCl with heparin (50 units per 100 ml)

4. PBS with Ca (II) and Mg (II) and heparin (50 units per 100 ml)

7. 30% BSA

10. Filters for cells with pores of 70 μm

11. 15 ml centrifuge tubes

**Steps:**

1. Collect the blood into BD Vacutainer® Heparin Tubes. Isolation of CTC should be done as soon as possible after collecting the blood!
2. Centrifuge 3 ml of blood (1,500 g x10 min), remove the plasma;
3. Rinse twice a 1ml-pipet tip with 30% BSA. The BSA treatment reduces adhesion of blood cells to plastic tips. Always use the BSA treated tips for cells!
4. Transfer the cell pellet into a 15 ml centrifuge tube using the BSA treated tip, rinse the Vacutainer with 2 ml of 0.42% NH_4_Cl with heparin, pipet several times and pour into the 15 ml tube, add the 8 ml of 0.42% NH_4_Cl with heparin into the 15 ml tube, incubate for 10 min on a shaker;
5. Centrifuge cells at 3,500 g for 5 minutes, remove the supernatant, add 3 ml of 0.2% NaCl with heparin to the pellet, resuspend the cells by pipetting and incubated for 60 minutes on a shaker. The 0.2% NaCl destroys the blood cell leaving cancer cells intact!
6. Centrifuge cells at 3,500 g for 5 minutes, remove the supernatant and add 3 ml of 0.9% NaCl with heparin, resuspend the cell pellet and leave in 0.9% NaCl for 10 min to recover the cell shape;
7. Centrifuge cells at 3,500 g for 5 minutes, remove the supernatant, add 1 ml of 0.9% NaCl with heparin to the pellet, resuspend the sample. Large flakes with mucus from the destroyed white blood cells will appear.
8. Filter the cells with a 70 μm-filter to a new 15 ml tube;
9. Add 1 ml of 0.9% NaCl with heparin to the pellet, resuspend and filter again;
10. Centrifuge at 3,500 g for 5 minutes, remove the supernatant and add 100 μl of PBS with heparin and 0.1 mg/ml yeast RNA to the pellet, incubate for 30 min on a shaker;
11. Add 4 μl of 500 nM aptamers and incubate for 10 min on a shaker in dark;
12. Centrifuge cells at 3,500 g for 5 minutes, remove the supernatant, transfer the cells to a glass slide, dry and fix the cells with 70% methanol for 5-10 min in dark;
13. Stain with Romanowsky-Giemsa dye.

**Table S1.** Characteristics of the tissues/blood/patients utilized at each round of aptamer selection to lung adenocarcinoma.

| **Round #** | **Individuals** | | | | **Tissues taken for selection** | |
| --- | --- | --- | --- | --- | --- | --- |
|  | *Diagnosis* | *Type* | *Age* | *Gender* | *Positive selection* | *Negative selection* |
| 1 | Cancer of the upper lobe of the right lung | AD T1N0M0 | 71 | male | Tumor tissue | - |
| 2 | Cancer of the lower lobe of the right lung | Poorly differentiated AD T4N2M0 | 51 | male | Tumor tissue | Normal tissue adjacent to the tumor |
|  | Healthy | - | 26 | male | - | Blood |
| 3 | Cancer of the left lung | Highly differentiated AD T2N0M0 | 77 | male | Tumor tissue | Normal tissue adjacent to the tumor |
|  | Healthy | - | 25 | male | - | Blood |
| 4 | Cancer of the upper lobe of the right lung | AD T2N0M0 | 53 | male | Tumor tissue | Normal tissue adjacent to the tumor |
|  | Healthy | - | 22 | female | - | Blood |
| 5 | Cancer of the upper lobe of the right lung | Moderately differentiated AD T2N0M0 | 65 | male | Tumor tissue | Normal tissue adjacent to the tumor |
|  | Healthy | - | 23 | female | - | Blood |
| 6 | Cancer of right lung cancer | high-grade AD T2N0M0 | 62 | female | Tumor tissue | Normal tissue adjacent to the tumor |
|  | Healthy | - | 25 | male | - | Blood |
| 7 | Peripheral cancer of the upper lobe of the right lung | AD T2N0M0 | 59 | female | Tumor tissue | Normal tissue adjacent to the tumor |
|  | Healthy | - | 25 | male | - | Blood |
| 8 | Cancer of the upper lobe of the right lung | Moderately differentiated AD T2N0M0 | 60 | male | Tumor tissue | Normal tissue adjacent to the tumor |
|  | Healthy | - | 26 | female | - | Blood |
| 9 | Cancer of the upper lobe of the right lung | Poorly differentiated AD T2N0M0 | 49 | male | Tumor tissue | Normal tissue adjacent to the tumor |
|  | Healthy | - | 23 | male | - | Blood |
| 10 | Peripheral cancer of the lower lobe of the right lung | Moderately differentiated AD T2N0M0 | 72 | female |  |  |
|  | Healthy | - | 25 | male | - | Blood |
| 11 | Cancer of the upper lobe of the left lung | AD T1N0M0 | 41 | female |  |  |
|  | Healthy | - | 22 | female | - | Blood |


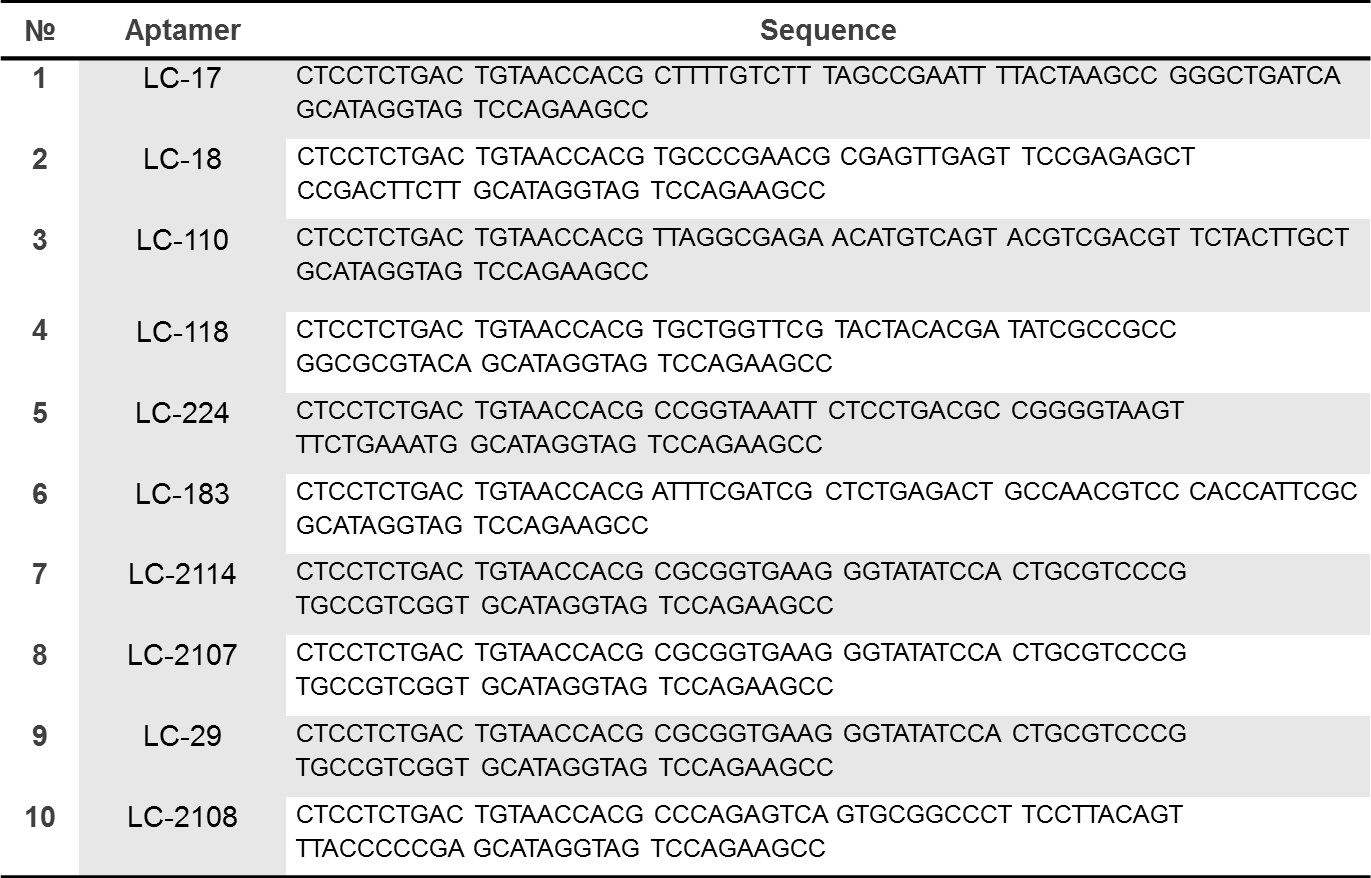
**Table S2**. Sequences of individual aptamers selected to postoperative lung adenocarcinoma tissues.

**Table S3.** Potential protein targets for aptamer LC-17

| Adenocarcinoma, patient 3 |  | Adenocarcinoma, patient 22 |  | Adenocarcinoma, patient 27 |  |
| --- | --- | --- | --- | --- | --- |
| Protein | Corr. coeff | Protein | Corr. coeff | Protein | Corr. coeff |
| 60 kDa heat shock protein, mitochondrial | 0.99 | Apolipoprotein A-II | 0.97 | Clusterin | 0.95 |
| Chromobox protein homolog 3 | 0.98 | Histone H2B | 0.83 | Vitronectin | 0.87 |
| Actin, cytoplasmic 1 | 0.98 | Vimentin | 0.81 | Anterior gradient protein 2 homolog | 0.81 |
| Heterogeneous nuclear ribonucleoprotein H | 0.97 | Lysozyme C | 0.74 | Tubulin alpha | 0.81 |
| Splicing factor, proline- and glutamine-rich | 0.97 | Actin-related protein 2/3 complex subunit 5 | 0.71 | Delta(3,5)-Delta(2,4)-dienoyl-CoA isomerase, mitochondrial | 0.79 |
| Transitional endoplasmic reticulum ATPase | 0.95 | Annexin A5 | 0.70 | Aldo-keto reductase | 0.73 |
| Histone H3.1; 3.3; 3.2; | 0.94 | Lumican | 0.69 | RuvB-like 2 | 0.72 |
| Vimentin | 0.93 | Cofilin-1 | 0.66 | Serum amyloid P-component | 0.71 |
| ATP synthase subunit beta, mitochondrial | 0.92 | Alpha-1-antitrypsin;Short peptide from AAT | 0.63 | Annexin A2 | 0.70 |
| ATP synthase subunit alpha, mitochondrial | 0.92 | Apolipoprotein E | 0.62 | Galectin-3-binding protein | 0.69 |
| Myosin-10 | 0.9 | Annexin A2 | 0.61 | Apolipoprotein A-I | 0.69 |
| Histone H2A | 0.89 | Phosphoglycerate kinase 1 | 0.56 | Neutrophil elastase | 0.69 |
| Tubulin alpha chain | 0.84 |  |  | Serotransferrin | 0.65 |
| Histone H1.0 | 0.82 |  |  | Alpha-1-antitrypsin | 0.64 |
| Histone H2B | 0.77 |  |  | Histone H4 | 0.59 |
| Lamin-B2 | 0.77 |  |  | Alpha-1-acid glycoprotein 1 | 0.57 |
| Heat shock protein beta-1 | 0.77 |  |  | Sorcin | 0.56 |
| Tropomyosin alpha-1 chain | 0.75 |  |  | Apolipoprotein C-I;Truncated apolipoprotein C-I | 0.56 |
| Tripeptidyl-peptidase 1 | 0.71 |  |  | Annexin A5 | 0.55 |
| Annexin A2; | 0.70 |  |  | Cathepsin D | 0.55 |
| Neutrophil defensin 1 | 0.70 |  |  | 60 kDa heat shock protein, mitochondrial | 0.54 |
| NHP2-like protein 1 | 0.69 |  |  | Gelsolin | 0.53 |
| Annexin A5 | 0.68 |  |  | Heat shock protein HSP 90-beta | 0.52 |
| Tubulin beta chain | 0.68 |  |  | Tubulin beta chain | 0.51 |
| Protein disulfide-isomerase A3 | 0.68 |  |  | Vimentin | 0.49 |
| Lamin-B1 | 0.67 |  |  |  |  |
| Protein PML | 0.67 |  |  |  |  |
| Histone H4 | 0.66 |  |  |  |  |
| Heterogeneous nuclear ribonucleoprotein F | 0.65 |  |  |  |  |
| Histone H2A.Z | 0.64 |  |  |  |  |
| Myosin-9 | 0.63 |  |  |  |  |
| Prelamin-A/C;Lamin-A/C | 0.57 |  |  |  |  |

**Table S4.** Potential protein targets for the aptamer LC-18

| Adenocarcinoma, patient 27 |  | Squamous cell, patient 23 |  | Adenocarcinoma, patient 17 |  |
| --- | --- | --- | --- | --- | --- |
| Protein | Corr. coeff | Protein | Corr. coeff | Protein | Corr. coeff |
| Lumican | 0.93 | Epiplakin | 0.97 | Fibrinogen beta chain | 0.62 |
| Actin-related protein 3 | 0.78 | Heterogeneous nuclear  ribonucleoprotein U | 0.96 | Histone H2B | 0.55 |
| Alpha-1-acid glycoprotein 1 | 0.70 | Heat shock protein HSP 90-beta | 0.92 | Histone H4 | 0.55 |
| Vitronectin | 0.68 | Prohibitin-2 | 0.92 | Histone H2A | 0.52 |
| Serotransferrin | 0.64 | Vitronectin | 0.91 | Neutrophil defensin | 0.51 |
| Tubulin alpha | 0.62 | Heat shock protein beta-1 | 0.81 | Fibronectin; Anastellin | 0.49 |
| Cathepsin D | 0.62 | Histone H2B | 0.77 |  |  |
| Histone H2B | 0.62 | Prohibitin | 0.76 |  |  |
| Anterior gradient protein 2  homolog | 0.52 | Tubulin beta-4B chain | 0.72 |  |  |
| Neutrophil defensin | 0.51 | Glyceraldehyde-3-phosphate  dehydrogenase | 0.70 |  |  |
| RuvB-like 2 | 0.50 | Glutathione S-transferase P | 0.69 |  |  |
| Transgelin-2 | 0.50 | Aldo-keto reductase | 0.66 |  |  |
| Tubulin beta | 0.49 | Plakophilin-1 | 0.63 |  |  |
| Glutathione S-transferase P | 0.47 | Cornifin-B;Cornifin-A | 0.60 |  |  |
| Aldo-keto reductase | 0.47 | Desmoplakin | 0.50 |  |  |
| Myosin light polypeptide 6 | 0.46 | Malate dehydrogenase,  mitochondrial | 0.47 |  |  |
| Apolipoprotein A-I; | 0.46 | 10 kDa heat shock protein,  mitochondrial | 0.47 |  |  |
| Glucose-6-phosphate  1-dehydrogenase | 0.46 | ATP synthase subunit beta,  mitochondrial | 0.47 |  |  |
|  |  | 60 kDa heat shock protein,  mitochondrial | 0.45 |  |  |
|  |  | Tubulin alpha | 0.45 |  |  |
|  |  | Alpha-enolase | 0.44 |  |  |
|  |  | Neutrophil defensin | 0.44 |  |  |

**Table S5.** Potential targets for aptamer LC-110

| Squamus cell, patient 4 |  | Adenocarcinoma, patient 22 |  | Adenocarcinoma, patient 23 |  |
| --- | --- | --- | --- | --- | --- |
| Protein | Corr. coeff | Protein | Corr. coeff | Protein | Corr. coeff |
| Myosin-9 | 0.97 | Grancalcin | 0.98 | Apolipoprotein A-II | 0.92 |
| Biglycan | 0.67 | Phosphoglycerate kinase 1 | 0.95 | RuvB-like 1 | 0.88 |
| Peroxiredoxin-1 | 0.66 | Clusterin | 0.94 | Tubulin alpha | 0.76 |
| ATP synthase subunit alpha, mitochondrial | 0.65 | ATP synthase subunit alpha, mitochondrial | 0.94 | Vitronectin | 0.76 |
| Histone H1.0 | 0.65 | Annexin A5 | 0.88 | Delta(3,5)-Delta(2,4)-dienoyl-CoA isomerase, mitochondrial | 0.69 |
| Histone H4 | 0.64 | Vimentin | 0.77 | Myosin-9 | 0.66 |
| ATP synthase subunit beta, mitochondrial | 0.61 | Coronin-1A | 0.76 | Clusterin | 0.64 |
| Histone H2B | 0.60 | Cathepsin G | 0.70 | Vitamin K-dependent protein S | 0.63 |
| Signal recognition particle 14 kDa protein | 0.58 | Complement component C9 | 0.70 | Complement C3 | 0.61 |
| Cofilin-1 | 0.58 | Peroxiredoxin | 0.69 | Apolipoprotein A-IV | 0.55 |
| Vimentin | 0.57 | Lumican | 0.69 | Lysozyme C | 0.54 |
| Pulmonary surfactant-associated protein | 0.57 | Annexin A3 | 0.69 | Neutrophil elastase | 0.50 |
| Lactotransferrin | 0.55 | Cytidine deaminase | 0.65 | Plasminogen;Plasmin heavy chain | 0.50 |
| Tropomyosin alpha | 0.55 | Ig gamma-1 chain C region | 0.63 | Glucose-6-phosphate 1-dehydrogenase | 0.49 |
| Histone H2A | 0.54 | Neutrophil elastase | 0.58 | Histone H2B | 0.49 |
| Annexin A2 | 0.49 | Pyruvate kinase isozymes M1/M2 | 0.58 |  |  |
| Histone H3.1 | 0.48 | Vitronectin | 0.57 |  |  |
| Serpin H1 | 0.48 | Apolipoprotein A-I | 0.55 |  |  |
| Clusterin | 0.46 | Histone H2B | 0.54 |  |  |

The target proteins were captured and purified from minced lung tumor tissues from patients with lung cancer using aptamer modified magnetic particles and identified using high performance liquid chromatography and high-resolution tandem mass spectrometry. All proteins in the table were identified by at least 3 peptides and quantitatively dominated over the samples with the control library in 3 independent experiments. Proteins were sorted by the decrease in correlation coefficients for the relative abundance in the aptamer samples vs. the control.


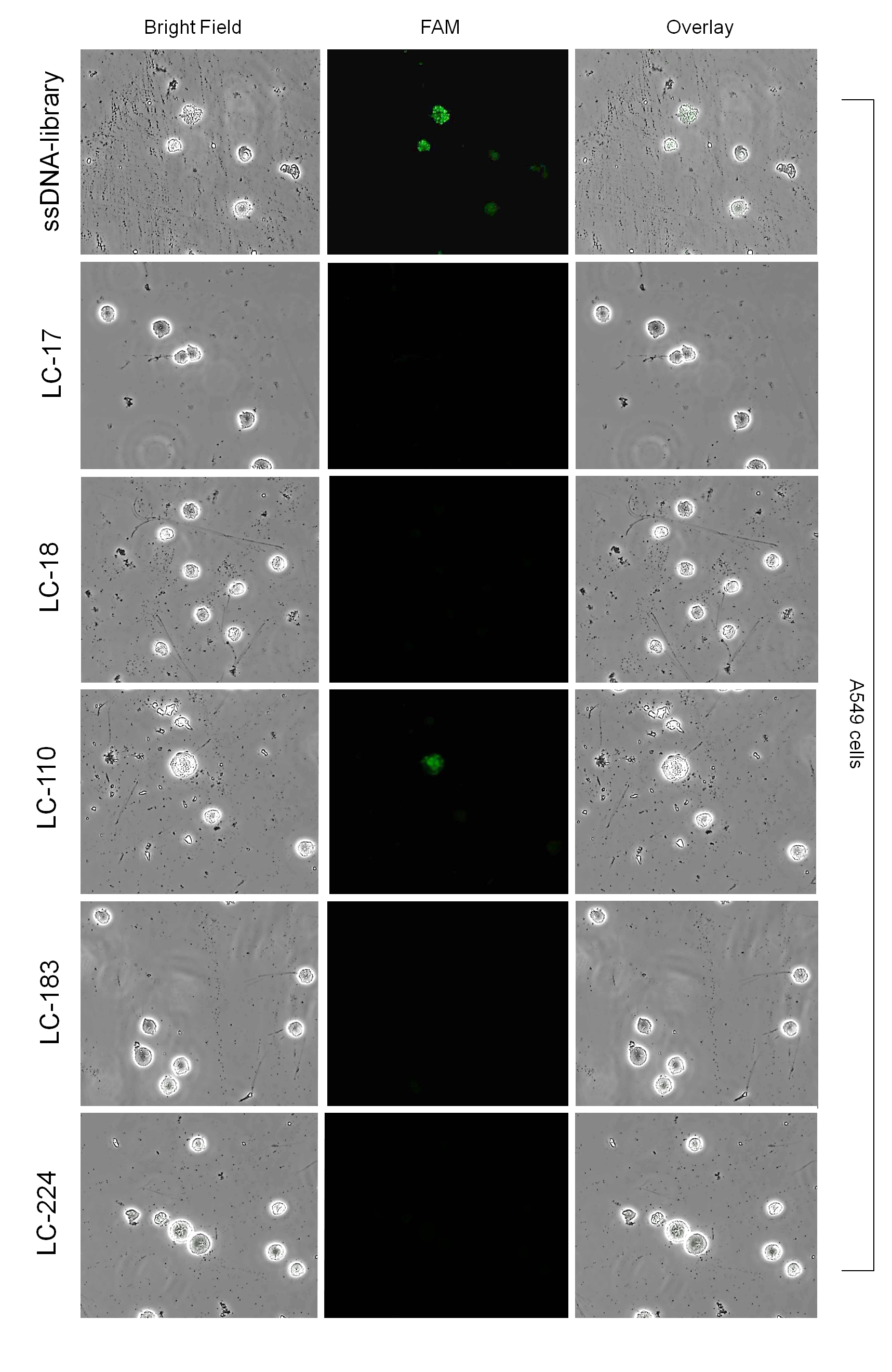


**Figure S1. Analyses of aptamer specificity to A549 cancer cell line.** Confocal microscopy analysis of staining A549 cultured cells with aptamer clones. The cells were pre-incubated with 0.1 mg mL^-1^ masking RNA for 20 min and then separately with 50 nM FAM-labeled LC-18, LC-17, LC-110, LC-183, LC-224, and ssDNA-library (a control) for 30 min at 25 °C, washed with DPBS once.


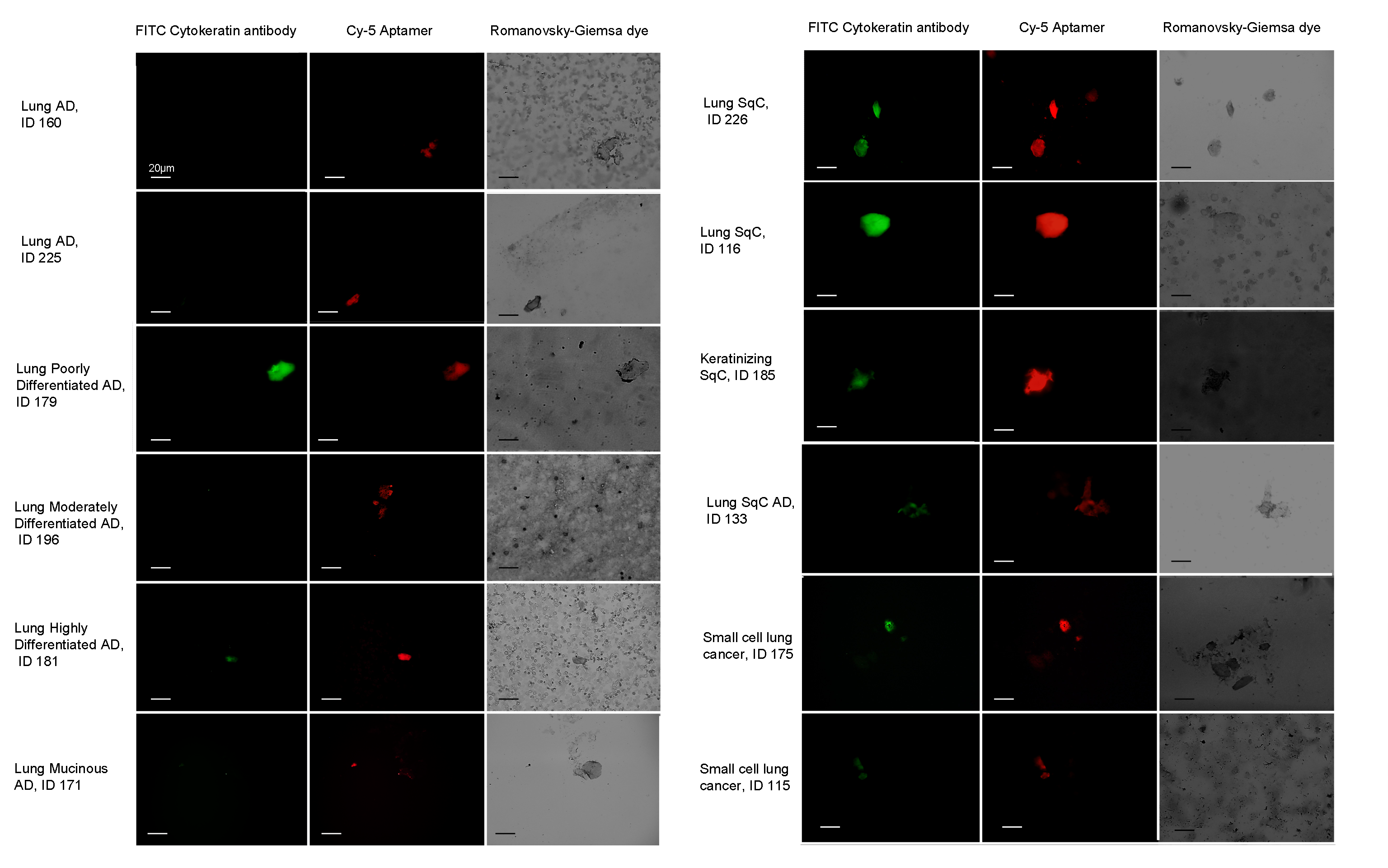


**Figure S2.** Fluorescent microscopy of blood smears of different lung cancer types. Samples were pre-incubated with masking DNA, Cy-5 labeled LC-18 and FITC-labeled anti-pan cytokeratin antibodies. The samples were spread evenly on a glass slide. The smears were fixed in methanol for 5 min and then stained with Romanowsky-Giemsa dye.


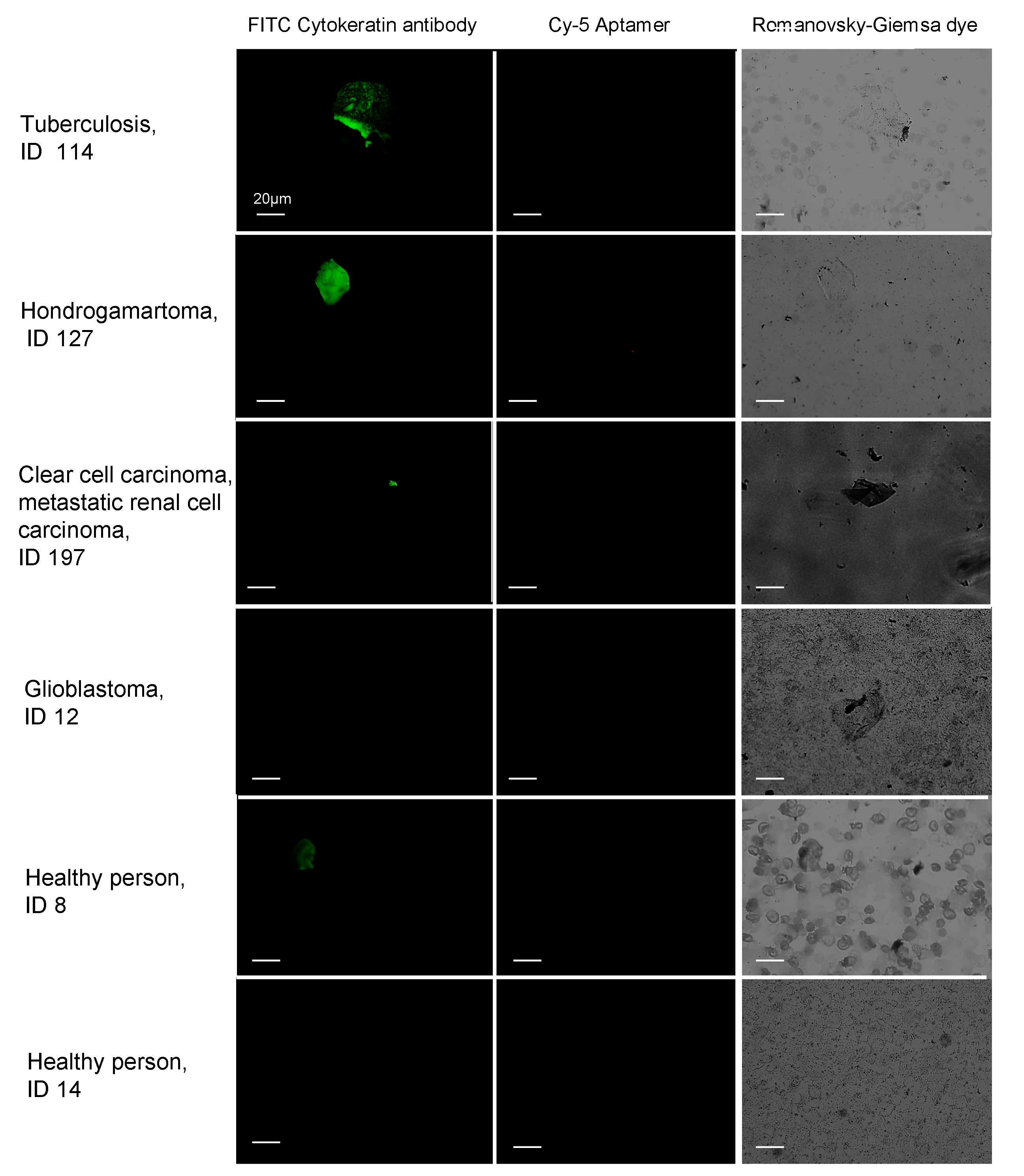


**Figure S3.** Fluorescent microscopy of blood smears of different not lung cancer other diseases and healthy people. Samples were pre-incubated with masking DNA, Cy-5 labeled LC-18 and FITC-labeled anti-pan cytokeratin antibodies. The samples were spread evenly on a glass slide. The smears were fixed in methanol for 5 min and then stained with Romanowsky-Giemsa dye.
